# Supplementary material for: Epigenetic modulation of RIPK3 by transglutaminase 2-dependent serotonylation of H3K4me3 affects necroptosis
Source: Cell Mol Life Sci. 2025 Apr 10;82(1):154. doi: 10.1007/s00018-025-05640-w (PMC11985880; doi:10.1007/s00018-025-05640-w)
Supplement: Supplementary file 1 — Supplementary Material 1 [file 18_2025_5640_MOESM1_ESM.docx]

**Figure S1.**

(A) RIPK3 mRNA levels, quantified by RT-qPCR in WT and TG2 KO MEFs in basal conditions normalized on actin levels. The graph shows the mean ± SEM of the mRNA levels from three independent experiments. The P-value was determined by unpaired Student’s *t* test (**p < 0.01). (B) Representative western blot analysis of RIPK3 in WT and TG2 KO MEFs in basal conditions. Actin was used as loading control. (C) The graph shows the mean ± SEM of the absorbance (OD 570 nm) of MTT assay from three independent experiments performed on WT and TG2 KO MEFs untreated and treated with TSZ for 4 h. The P-value was determined by two-way ANOVA with Sidak’s multiple comparation test (***p< 0.001).

**Figure S2.**

(A) Representative images and related graph showing the percentage of TUNEL positive cells in WT MEFs and TG2 KO MEFs after treatment with TNFα for 16h and 24h. The graph shows the mean ± SEM of the percentage of positive cells from three independent experiments. The P-value was determined by two-way ANOVA with Sidak’s multiple comparation test (****p< 0.0001). (B) Representative western blot analysis of Caspase 8 in TG2 KO MEFs after TNFα treatment. Actin was used as loading control. The graph shows the mean ± SEM of densitometric analysis from three independent experiments. The P-value was determined by unpaired Student’s *t* test (**p < 0.01).

**Figure S3.**

(A) Protein-protein interaction network of the DNA methylases and demethylases identified in the TG2 nuclear interactome analysis. The network, containing identiﬁed proteins, was mapped using the STRING system (http://string-db.org/). (B) Representative western blot of RIPK3 in WT MEFs and TG2 KO MEFs treated with different concentrations of AZA for 4 days. Actin was used as loading control. (C) Representative western blot and densitometric analysis of RIPK3 in TG2 KO MEFs treated with AZA 20 μM for 4 days. Actin was used as loading control. The graph shows the mean ± SEM of densitometric analysis from three independent experiments. The P-value was determined by unpaired Student’s *t* test (*p < 0.05). (D) Representative western blot and densitometric analysis of RIPK1 and caspase 3 in TG2 KO MEFs treated with AZA for 4 days and with TNFα for 16 and 24h. Actin was used as loading control. The graph shows the mean ± SEM of densitometric analysis from three independent experiments. The P-value was determined by unpaired Student’s t test (*p < 0.05, **p < 0.01, ***p< 0.001 ****p< 0.0001).
